# Assessing fecal load with ultrasound in children with colorectal pathology: ReKiSo study

**DOI:** 10.1007/s00383-024-05771-4

**Published:** 2024-07-19

**Authors:** Daniel Erkel, Stefanie Märzheuser, Judith Lindert

**Affiliations:** Department of Pediatric Surgery, University Hospital Rostock, Ernst-Heydemann-Straße 8, 18057 Rostock, Germany

**Keywords:** Point-of-care ultrasound, Constipation, Transabdominal ultrasound, Transrectal diameter, Bowel management, Pediatric colorectal disease, Fecal load

## Abstract

**Purpose:**

To evaluate bowel management for children with colorectal pathology by measuring transverse rectal diameter (TRD) and assessing fecal load with transabdominal rectal ultrasound (TRU).

**Methods:**

Prospective case–control study of children receiving bowel management (BM) between 04/2023 and 04/2024 was done. There was inclusion of patients with Hirschsprung disease (HD), anorectal malformation (ARM) and functional constipation (FC). Patients with other congenital or neurological conditions were excluded. Control group consisted of inpatients and outpatients without abdominal complaints. FC was diagnosed according to ROM-IV-criteria. For HD and ARM, we followed a list of symptoms. To assess fecal load, we visualized the TRD using the Klijn (Klijn et al. in J Urol 172:1986–1988, 2004) method. The bladder was moderately full. The fecal load was assessed retrograde from the rectum. Follow-up was at 1/3/6 months. Secondary data were collected from medical records. Sample size calculated a priori and follow-up group with new gathered data.

**Results:**

*p* value for TRD in all groups significant with *p* < 0.05 and in grouped follow-up.

**Conclusion:**

Ultrasound is a useful tool for assessing fecal load and helps diagnose constipation and monitor BM. Irrespective of colorectal pathology, a cut-off of 3 cm seems to discriminate between children without constipation/overload symptoms and asymptomatic patients. We present a radiation-free method for monitoring bowel management.

## Introduction

Anorectal malformation (ARM) and Hirschsprung disease (HD) are rare congenital colorectal disorders. Despite surgical treatment in the form of posterior/anterior sagittal anorectoplasty (PSARP/ASARP) or pull-through surgery, many of these patients suffer from constipation, stool soiling and problems of defecation [1]. The follow-up concentrates on bowel management (BM) to achieve social cleanness and continence [2, 3]. During BM, the fecal load of the colon is monitored by daily abdominal x-rays for a week [4, 5]. The long-term effects of repetitive exposure in children to radiation are described by Linet [6], and Wall [7] estimated the highest risk for any kind of cancer in the age group < 10 years using the Monte Carlo method, which is the primary target group for this kind of intervention and follow-up. Overall, the smaller height, slight reduction and increased scattered radiation result in a higher absorbed doses [6].

In addition, functional constipation (FC) is the main cause of constipation and a common reason for admission to the emergency department [8]. For diagnosis or evaluation of fecal load abdominal, X-rays are used in 70–77.5% of the cases, although the utility is considered low [9], lacks medical evidence [10] and ESPGHAN/NASPGHAN has not included X-ray in their recommendation [11]. Furthermore, the benefit is questionable due to low sensitivity, specificity, diagnostic accuracy and simply subjective assessment. There is no standardized evaluation and scores by Barr, Leech or stool loading show low interobserver reproducibility [12, 13]. In particular, stool loading correlates poorly with the symptoms of constipation and even an unremarkable X-ray does not represent a normal finding or the exclusion of a serious disease [14, 15]. In fact, the most common missed diagnoses include acute appendicitis and intussusception [12, 16]. Otherwise, a readmission leads to overdiagnosis [16]. This should be put into consideration because patients with constipation show more readmissions and patients with ARM or HD need a prolonged BM [12].

Recent research presents abdominal ultrasound examination as an alternative diagnostic tool for FC and evaluation of treatment. In comparison to abdominal radiographs, this is an accurate modality and would avoid X-rays despite established scores [17] or charting [18]. Ultrasound is non-invasive and has no adverse effect. This simple procedure should make a digital rectal examination (DRU) obsolete [19, 20]. Furthermore, not inconsiderable costs for unjustified X-ray requests can also be saved in these cases [17] and the principle of ALARA (as low as reasonably achievable) satisfied. Unfortunately, there are no data regarding ARM or HD and those patients are consequently excluded in the current research. The aim of the ReKiSo Study (German: Rektum Kinder Sonographie) is to provide new data for these patient groups using established sonographic methods.

## Methods and patients

### Literature research and data extraction

Before setting up the study design, we performed literature research on scientific articles exploring the utility of ultrasound in children with FC. We used several keywords (i.e., transabdominal ultrasound, transrectal diameter, constipation) on pubmed.gov and found 15 articles to put into consideration. Klijn [21] described first a new method of abdominal ultrasound as a diagnostic tool for constipation in children with dysfunctional voiding by measuring the transrectal diameter (TRD) in the transverse plane. The following scientific research adapts Klijn’s method and faces new questions (i.e., position of probe, bladder filling, influence of treatment). In general, the inclusion of patients follows the ROM-criteria or an equivalent list of symptoms for chronic constipation. All studies excluded children with ARM or HD. Our key findings are summarized in Table 1 [17, 19, 21–33] and the complete table is attached to the appendix.

**Table 1:** Key findings literature research

Author, year	Number of study participants	TRD in cm	*p* value
Klijn et al. [21]	*N* = 49	mean_case_: 4.9 (SD 1.101) mean_control_: 2.1 (SD 0.64)	*p* < 0.001
Singh et al. [22]	*N* = 177	median_case_: 3.4 (2.1–7.0 with IQR 1.0) median_control_: 2.4 (1.3–4.2 with IQR 0.72)	*p* < 0.001
Bijoś et al. [23]	*N* = 225	Results for all subgroups by age: mean_case_: 4.3060 ± 0.968 mean_control_: 3.183 ± 0.824	*p* < 0.001
Joenssons et al. [19]	*N* = 51	Results pre-treatment: mean_case_: 3.96 ± 0.82 mean_control_: 2.14 ± 0.6	*p* < 0.001
Di Pace et al. [24]	*N* = 270	mean_case_: 3.9958 ± 0.6906 mean_control_: 1.0 ± 0.8319	*p* < 0.0005
Karaman et al. [25]	*N* = 66	Results pre-treatment: mean_case_: 3.42 ± 1.04 (full bladder) mean_control_: 2.12 ± 0.65 (full bladder)	*p* < 0.001
Modin et al. [26]	*N* = 28	mean_case_: 3.5 mean_control_: 1.9 (SD 0.3)	–/–
Hatori et al. [27]	*N* = 100	median_case_: 3.53 median_control_: 2.0	*p* < 0.0001
Doninger et al. [17]	*N* = 50	mean_case_: 4.3 ± 1.35 (IQR = 1.52) mean_control_: 2.85 ± 1.16 (IQR = 1.63)	Statistically significant
Momeni et al. [28]	*N* = 76	mean_case_: 3.172 ± 0.963 mean_control_: 1.985 ± 0.437	*p* < 0.001
Pop et al. [29]	N = 65	Results for all subgroups by age mean_case_: 3.59 ± 1.41 mean_control_: 2.42 ± 0.71	*p* < 0.05
Imanzadeh et al. [30]	*N* = 154	Results pre-treatment: mean_case_: 3.879 ± 1.017	*p* < 0.001
Doğan et al. [31]	*N* = 304	mean_case_: subgroups by age mean_control_: subgroups by age	*p* = 0.04 (group 3.1–6 years), * p* = 0.003 (group 6.1–12 years)
Hamdy et al. [32]	*N* = 100	median_case_: 3.55 (3.2–4) median_control_: 2.3 (1.8–2.5)	–/–
Gatzinsky et al. [33]	*N* = 110	mean_case_: subgroups by age mean_control_: subgroups by age	Not statistically significant

The researchers present data of TRD which was significantly higher (*p* < 0.05) in the case group compared to the control group for each study except in the age group < 1 year [33] and < 3 years [31]. Three studies [17, 22, 27] calculated a cut-off for constipation at 2.7–3.8 cm. The sensitivity varied between 71 and 100% and specificity between 71 and 97% [17, 25, 27, 32].

To evaluate TRD in case and control groups, we focused on seven studies with the specification of a mean value [17, 19, 21, 23–25, 29]. To assume the reduction of TRD in the follow-up, we orientated on five studies [17, 19, 25, 29, 30]. For estimation of the TRD in the case (ARM, HD and FC) and control groups, the median (3.16 cm) between mean TRDcase = 4.0674 cm and TRDcontrol = 2.259 cm of the reviewed studies was calculated. This median exceeded the 95% confidence interval of the mean difference = 1.82 cm (1.07–2.56). Thus, there is no overlap of the case and control groups. We concluded a minimum mean TRDcase of > 3.16 cm contrary to < 3.16 cm in the control groups as well for the case groups in the follow-up. The mean reduction of TRD in the follow-up was 1.26 cm and exceeded the calculated limiting value of 3.16 cm. Following the findings of Gatzinsky [33], TRD should not be useful for children < 1 year old.

### Study design

**Fig. 1:**
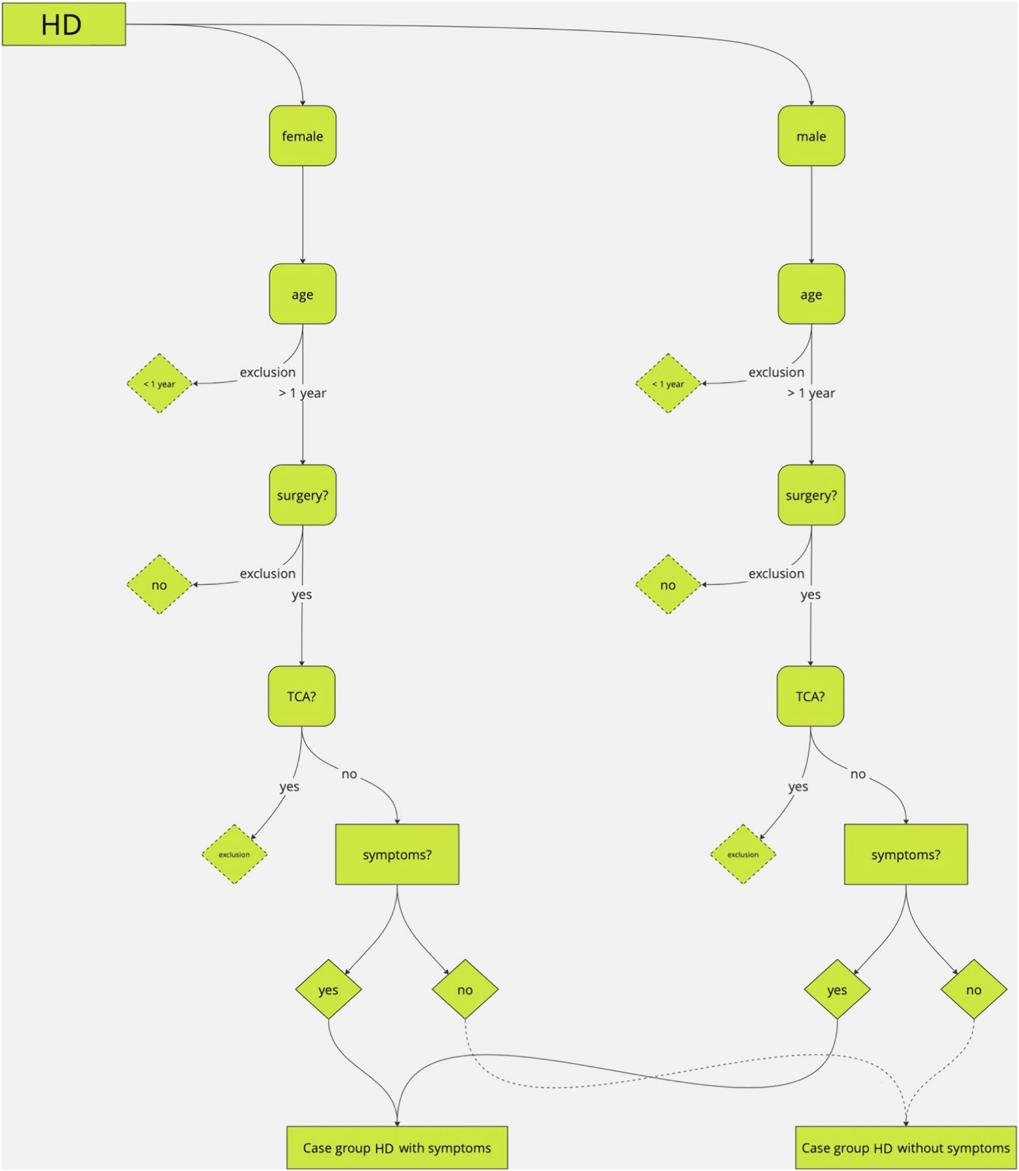
Process of in-/exclusion

**Fig. 2:**
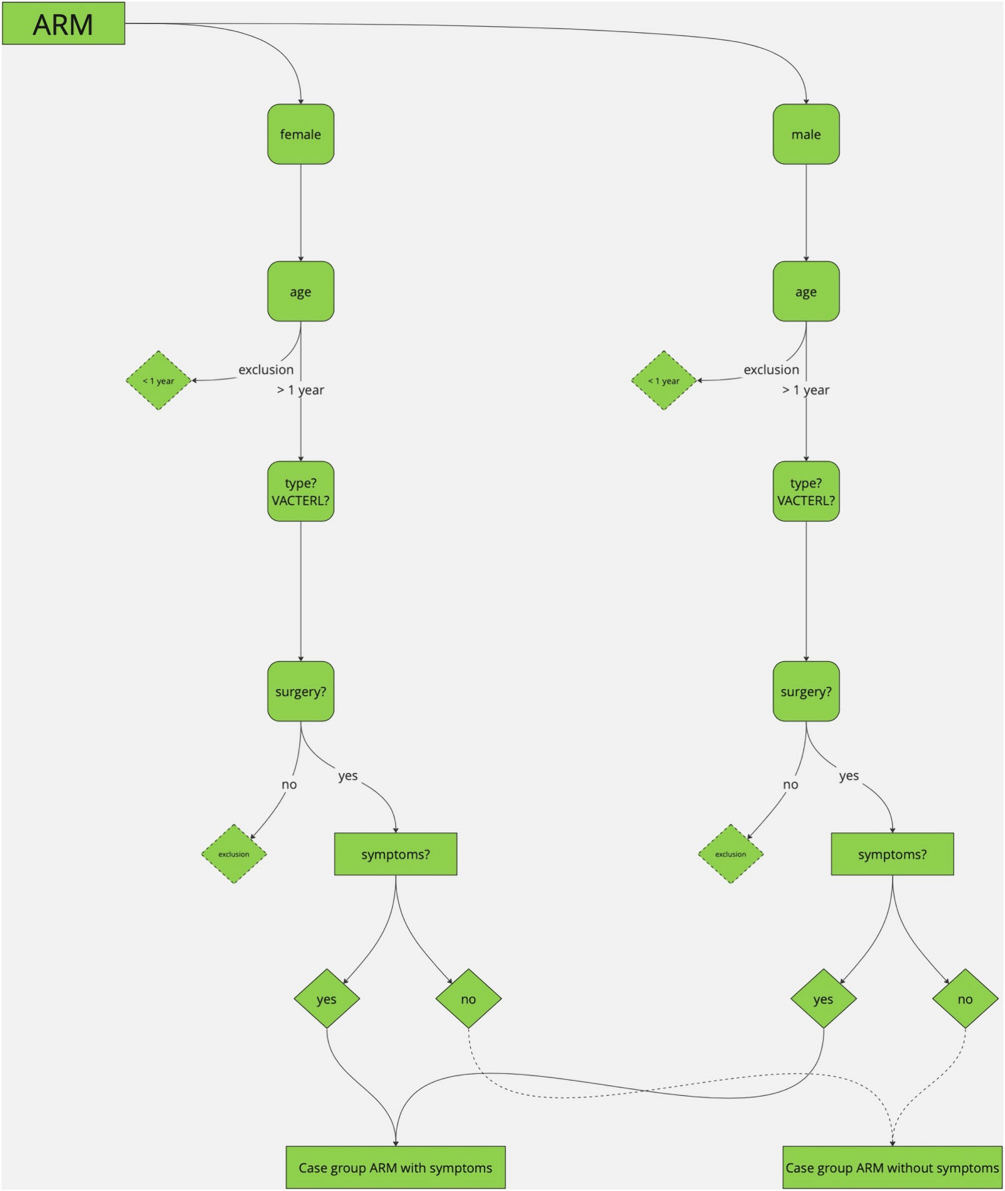
Process of in-/exclusion

ReKiSo is a prospective case–control study at the Clinic for Paediatric Surgery of the Rostock University Medical School (UMR) enrolling 302 children over a period of one year (04/2023—04/2024). We included 155 patients with ARM, HD or FC. Patients with other congenital anomaly affecting bowel function (ileal atresia, gastroschisis, omphalocele, cloacal exstrophy) or neurological condition (cerebral palsy, spina bifida, tethered cord) were excluded in the present analysis, but they were also monitored and will be reported separately. The control group consisted of children without a gastrointestinal pathology and without clinical signs of constipation, which were either hospitalized children or consulting the outpatients department for other reasons (trauma, urological or other pathology). FC was diagnosed by ROM-IV criteria [34] (see Table 2). For children with HD and ARM, we followed a list of symptoms as shown in Table 2. Secondary data (age, weight, height, comorbidity, surgery, transanal irrigation, clinical classification) were collected from medical records. The consultation in the special pediatric outpatient clinic for colorectal diseases included a careful anamnesis, clinical examination and finally the abdominal ultrasound scan. To our knowledge, we were the first to present data about TRD of children with ARM or HD. Therefore, the process for in- and exclusion is displayed in the following Figs. 1 and 2.

**Table 2:** ROM-IV-criteria and list of symptoms

ROM-IV-criteria for FC	List of symptoms for ARM and HD
- Min. 2 criteria for children > 4 years, duration > 1 month: ≤ 2 defecation/week, ≥ 1 period of incontinence/week, excessive stool retention, painful or hard bowel movements, large fecal mass in the rectum, large diameter stools that can obstruct the toilet - Both criteria for children < 4 years: ≥ 1 period of incontinence /week and large diameter stools that can obstruct the toilet only after development of cleanliness	- Constipation - Fecal incontinence - Painful defecation - Extensive defecation - Fecalith/filled bowel loop palpable - Stool in DRU - Enlarged/protruding abdomen - Meteorism

### Ultrasound

**Fig. 3:**
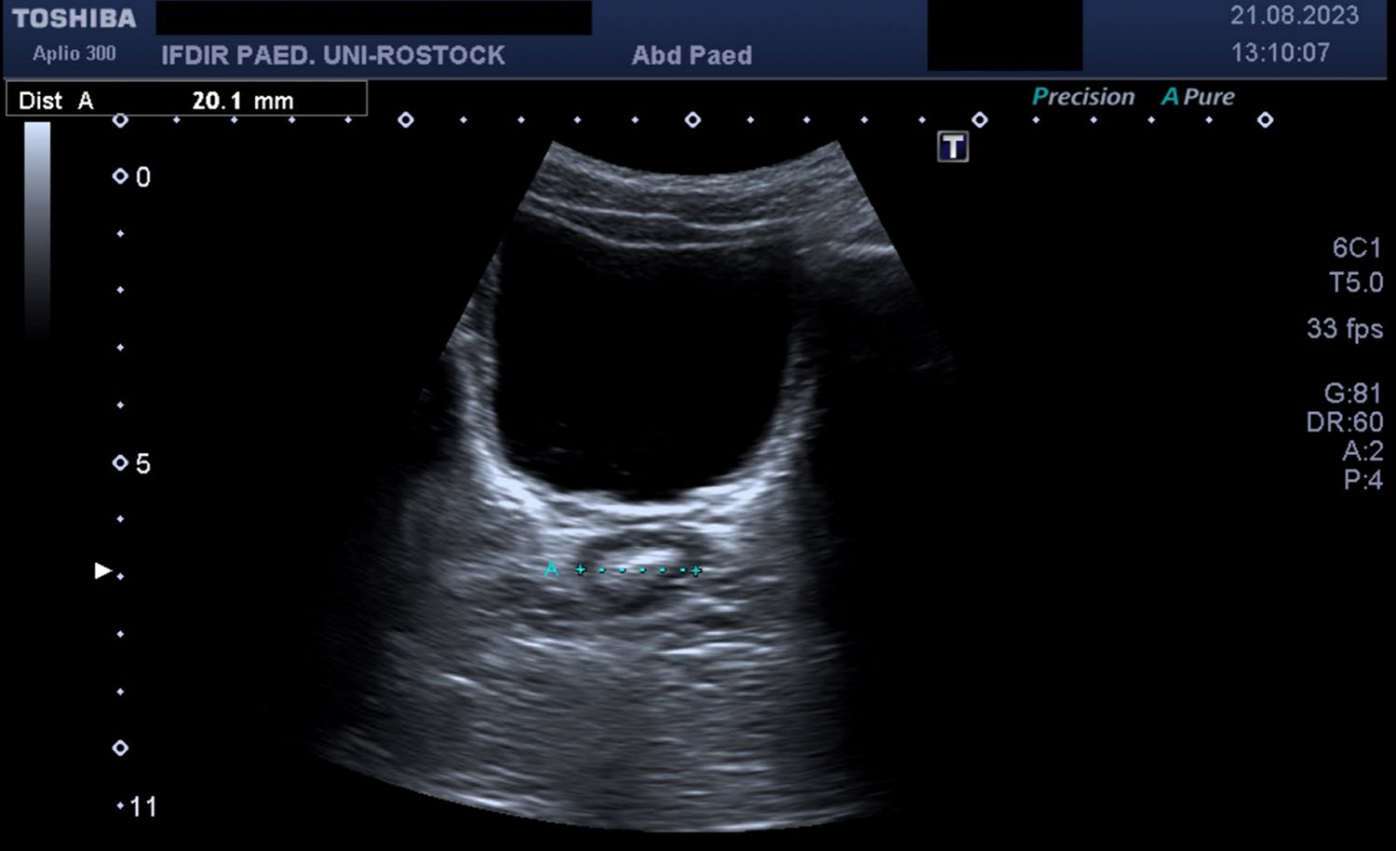
Asymptomatic patient with oval shaped and empty rectum

**Fig. 4:**
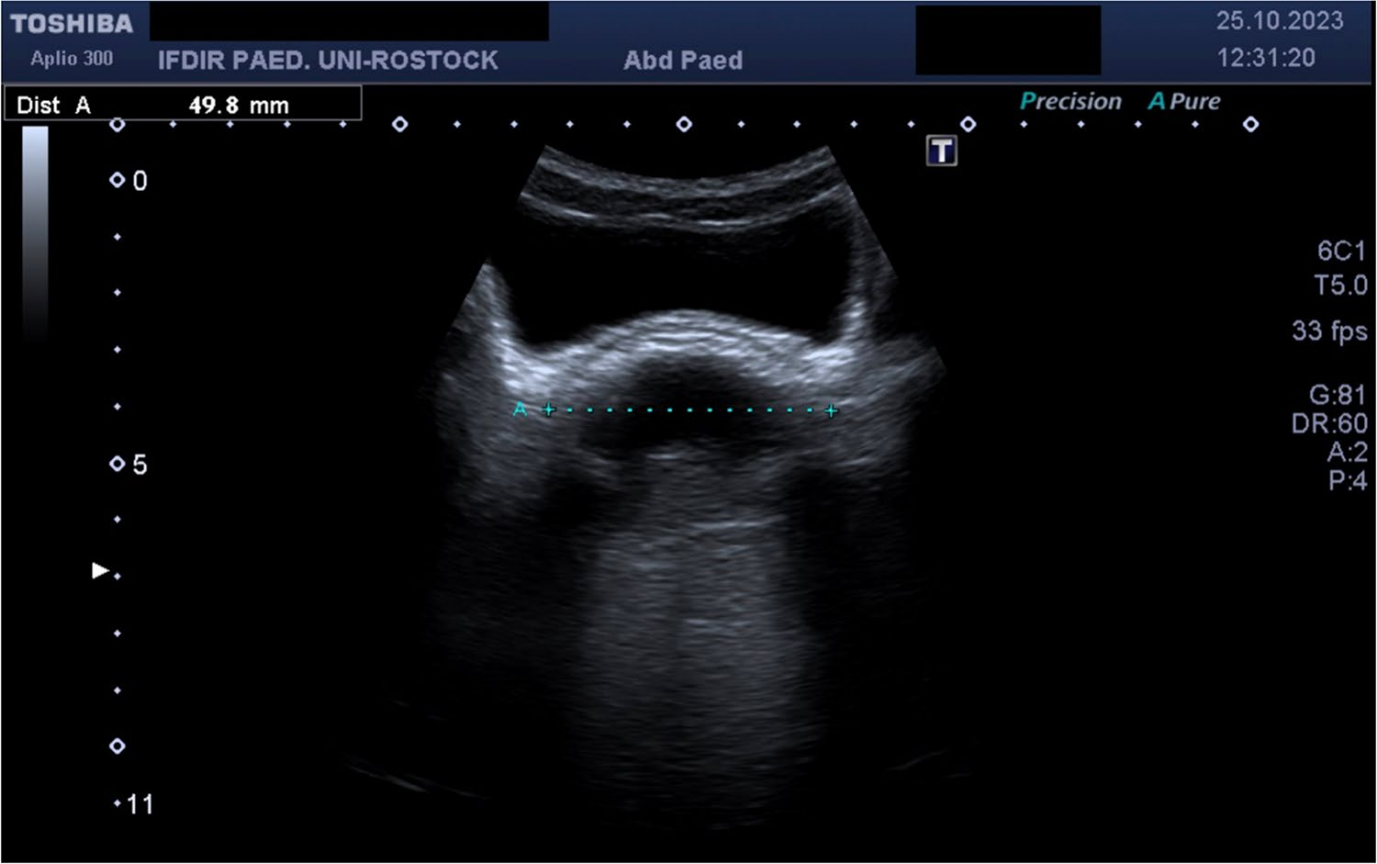
Constipated patient with hyperechoic crescent and posterior shadowing

We used the method of Klijn [21] by placing a curved array of 3.5 MHz (Toshiba Aplio 300, Toshiba Medical Systems GmbH, Germany, Neuss) above the symphysis and measured the largest TRD at a downward angle of at least 15 degrees from the transverse plane after distinguishing sigma from rectum. In constipated children, the rectum conducted as an adynamic structure and physiologically the ampulla recti as well the neorectum was empty. Thus, it was not necessary to determine TRD several times. The bladder was moderately full and acted as an acoustic window. We assessed fecal load retrograde starting at the rectum. Figs. 3 and 4 show typical measurements of TRD for asymptomatic and constipated patients. The follow-up was performed after 1/3/6 months, but readmission was possible at any time. Several patients were monitored with tele-medicine until reaching the clinical outcome (absence of symptoms) due to long distance. We treat children from all over Germany and neighboring countries in the patient cohort, but we lost final ultrasound measurements in their follow-up. The ultrasound scans were performed by one consultant and one medical student under her supervision.

### Statistics

Data management in our clinical patient registry on excel used pseudonymized acronyms. Statistical analyses were performed with SigmaPlot 13.0 (Inpixon GmbH, Germany, Düsseldorf) for descriptive statistics and verification of normal distribution by Shapiro–Wilk test. The* t*-test was used for normal distributed continuous values and otherwise the Mann–Whitney-*U*-Test. *p* value < 0.05 was statistically significant. Cut-offs, sensitivity and specificity were calculated using receiver operating characteristic. Non-linear correlation was computed for correlation in secondary data. To determine the number of cases in each group, G*Power 3.1.9.6 (Faul, Erdfelder, Lang & Buchner, 2007) [35] was operated for power analysis a priori with setting *α* = 0.05 and effect size *d* = 0.80. The sample size was a minimum of *n* = 42 for each case and control group. In the follow-up, a total sample size of *n* = 8 were calculated using the new gathered data. For this purpose, ARM and HD were assembled as a coherent group. For evaluation of the follow-up, the paired *t*-test was used.

### Ethical approval

This study was approved by the ethic committee for the Medical School of Rostock (A 2023-0066; 18.04.2023) and conducted in conformity to the Declaration of Helsinki. Informed consent was obtained from parents or legal guardians.

## Results

**Fig. 5:**
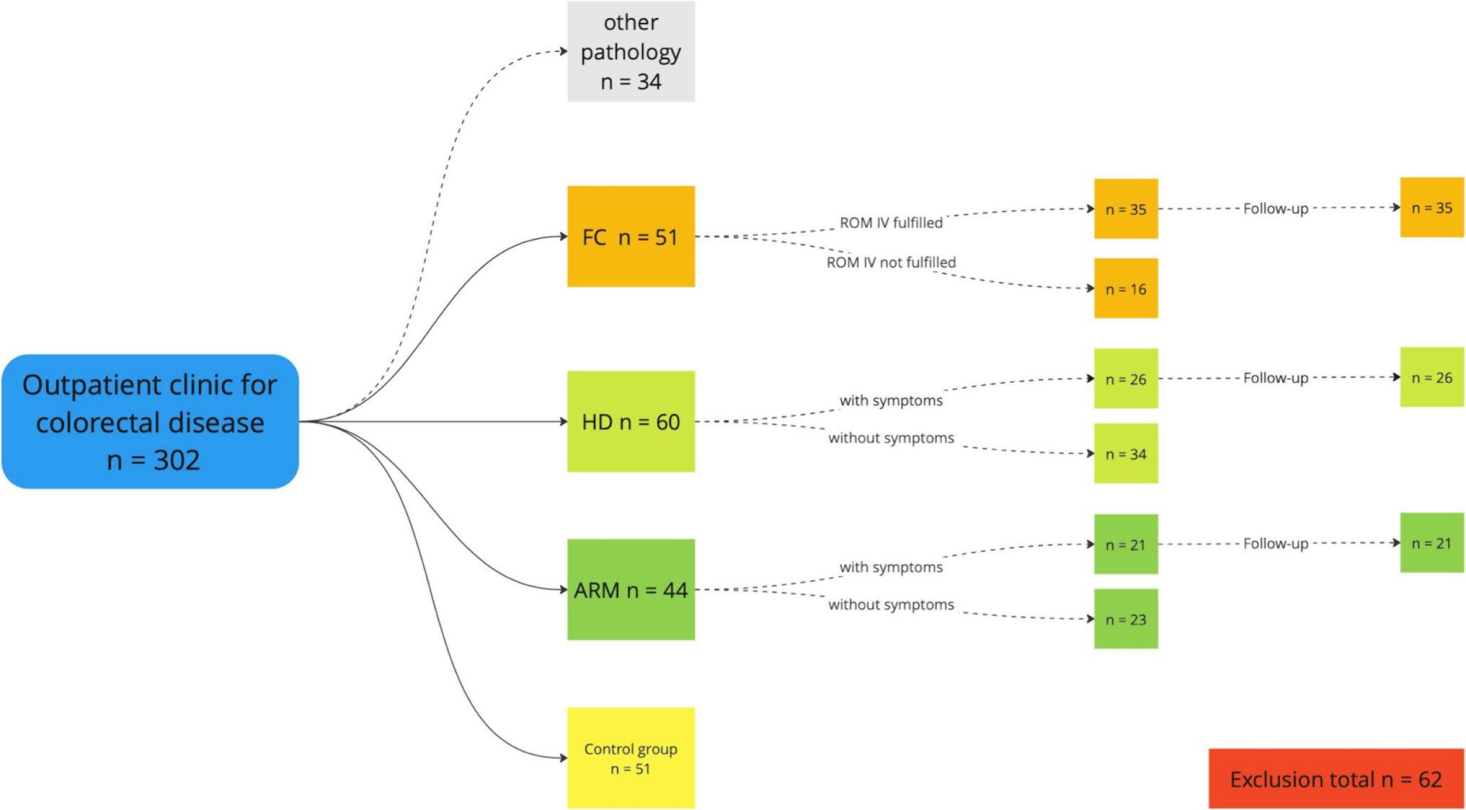
Overview of included patients

For one year, we performed ultrasound scans on 302 children and Fig. 5 shows the composition of groups considering the process of in- or exclusion. Patient’s characteristics are summarized in Table 3. In the first stage of the study, we investigated particularly the utility of ultrasound for children with colorectal pathology. We found significantly increased TRD for children with ARM or HD due to constipation, fecal soiling and associated symptoms. These findings conducted similar to published data of the literature research based on children with FC following ROME-IV-criteria.

**Table 3:** Patient’s characteristics

Group	Control group	HD	ARM	FC
Number	51	60	44	51
Sex (female:male)	15:36	13:47	24:20	26:25
Age (years, mean, SD)	7 (3.72)	5 (3.95)	6 (3.28)	7 (3.76)
Height (cm, mean, SD)	123.23 (24.73)	105.00 (26.44)	107.23 (21.5)	118.85 (26.43)
Weight (kg, mean, SD)	27.84 (14.18)	18.85 (11.47)	18.27 (6.54)	29.55 (19.10)

The control group was used for comparison to all case groups and faced problems considering demographic or physical characteristics. Generally, sex in patients with HD showed a ratio of 1:4 while ARM was balanced. There was no significant difference considering sex only in HD and the control group (*p* = 0.353). On the other hand, age was not statistically different for ARM and FC compared to the control group (*p* = 0.067 and *p* = 0.53) fading the sex difference. Only in FC height and weight were not statistically significant (*p* = 0.595 and *p* = 0.933).

The fecal load was assessed retrograde from the rectum. The ampulla recti was empty in all patients of the control group. Although patients in the case groups reported any symptoms, there was stool present in other parts of the colon ranging from 8.82 to 18.75%. The fecal load increased respectively presenting symptoms according to Table 2 for these groups from 71.43 to 91.43%. The correlation between TRD and fecal load was high and statistically significant (*p* < 0.0001). The correlation between demographic and physical characteristics was overall not statistically significant. The receiver operating characteristic was used for calculating cut-offs, sensitivity and specificity in each case group. The computed values are shown in Table 4.

**Table 4:** Sensitivity, specificity and cut-off in case groups

Group	MH	ARM	FC
Sensitivity	97.60%	95.65%	100%
Specificity	68.00%	57.14%	85.71%
Cuf-off (cm)	2.975	3.095	2.96

**Fig. 6:**
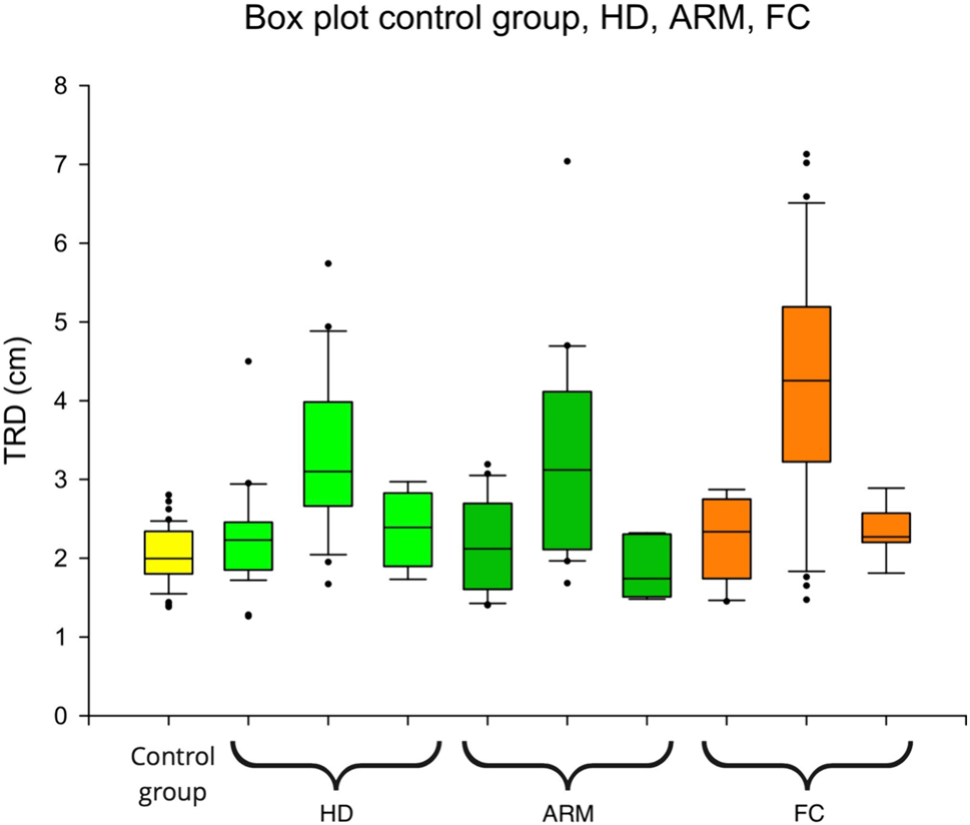
Box plot control group, case groups in the sequence of ‘no symptoms, symptoms and follow-up’ for HD, ARM and FC

The second stage of the study focused on the follow-up with BM and 82 children with symptoms or FC were included. The treatment covers laxatives, enemas or transanal irrigation and patients were evaluated after 1/3/6 months. The clinical endpoint was the absence of any signs of constipation according to ROM-IV-criteria or our list of symptoms (22 patients), see Table 2. The following Table 5 summarizes both stages of the study and the measured values are shown in Fig. 6.

**Table 5:** Summary

Group	Number of participants	Mean_TRD_ in cm	*p* value
Control	*N* = 51	2.049 (SD 0.368)	–/–
ARM	*N* = 44	- Without symptoms: 2.169 (SD 0.592) - With symptoms: 3.308 (SD 1.304) - Follow-up: 1.874 (SD 0.405)	- *p* < 0.001* - Follow-up* p* = 0.011*
HD	*N* = 60	- Without symptoms: 2.314 (SD 0.573) - With symptoms: 3.348 (SD 1.006) - Follow-up: 2.490 (SD 0.514)	- *p* < 0.001* - Follow-up *p* = 0.0382**
FC	*N* = 51	- ROM-IV not fulfilled: 2.310 (SD 0.505) - ROM-IV fulfilled: 4.357 (SD 1.382) - Follow-up: 2.476 (SD 0.362)	- *p* < 0.001* - Follow-up *p* < 0.001*

* = Mann–Whitney *U* test, ** = *t*-test

### Exclusion

During the survey, 34 patients with constipation and fecal soiling or associated symptoms were admitted in the pediatric outpatient clinic for colorectal diseases and ultrasound scans were performed. These patients had other congenital anomaly affecting bowel function (esophageal and ileal atresia, gastroschisis, omphalocele) or neurological condition (cerebral palsy, spina bifida, tethered cord). They were excluded in the present analysis, but they were additionally monitored and will be reported separately. Additionally, 62 patients of the case groups and control groups were finally excluded. Detailed exclusion is commented on below.

MH: In general, children after surgical treatment were included (pull-through surgery), but those with TCA were excluded in this analysis due to the total resection of the colon and inconsistent data. Following ROM-IV-criteria, patient groups younger or older than 4 years were investigated and finally patients younger than 1 year were excluded due to insufficient data.

ARM: In general, children after surgical treatment were included (PSARP/ASARP), but those without surgical treatment or special forms of ARM (i.e., atypical form, cloacal exstrophy) were excluded due to limited sample size. Following ROM-IV-criteria, patient groups younger or older than 4 years were investigated and finally patients younger than 1 year were excluded due to insufficient data.

FC: The main inclusion criterion was ROM-IV and some patients received surgical interventions of the abdomen in the past (i.e., appendectomy, rectal biopsy). Following ROM-IV-criteria, patient groups younger or older than 4 years were investigated and finally patients younger than 1 year were excluded due to insufficient data.

## Discussion

Patients with ARM or HD often present symptoms of constipation, stool soiling and problems of defecation during long-term follow-up. Sufficient BM is essential in the follow-up to achieve social cleanliness. Monitoring BM for these patient groups widely focused on abdominal X-rays [4, 5]. While ultrasound imaging for patients with FC was developed for diagnosing and the evaluation of BM in current research, these patients with colorectal pathology were excluded consistently.

The first part of the study focused on the utility of TRD to discriminate asymptomatic patients from patients with constipation, fecal incontinent or associated symptoms in ARM and HD. The current analysis of the study presented statistically significant data of TRD according to present data of FC and exceeded their cut-offs. The calculated cut-offs were similar to those of FC. Fecal load increased and was found retrograde in the colon. The sensitivity of abdominal ultrasound for ARM and HD was equivalent to FC. Specificity was slightly lower compared to data of FC because there was no score used and the study followed a list of symptoms. Additionally, patients with ARM reported more stool soiling while patients with HD were more likely to be constipated. However, the collective sensitivity of 95.89% and specificity of 72.84 for ARM, HD and FC were equivalent to values in the current research. The calculated cut-off for ARM and HD was 2.975 cm, but we suggest a practical cut-off of 3 cm for patients older 1 year.

During follow-up, TRD for ARM and HD was statistically significant lower as endpoints reached and conducted comparable to patients with FC. The options of treatment were identical for all case groups and FC was also scanned with abdominal ultrasound to exclude environmental effects. Thus, ultrasound imaging is an easy, non-invasive and not harmful tool for monitoring BM in patients with ARM and HD.

### Limits

The control group was used for three case groups with statistical differences. Although TRD was not affected, two individual control groups for each ARM and HD may be helpful to investigate further correlation or influence of secondary data. Due to tele-medicine, ARM and HD were grouped to reach the calculated sample size in the follow-up, but TRD was already statistically significant lower.

## Data Availability

For request of the research data, please contact the corresponding author.

## Appendix

For access of the complete table for the literature research (Table 1), please contact the corresponding author.
